# Effects of neighborhood level health and environment quality on academic and executive abilities in youth with Noonan syndrome spectrum disorder

**DOI:** 10.1017/S1355617726101969

**Published:** 2026-05-06

**Authors:** Sara Katharine Pardej, Odeya Russo, Tamar Green

**Affiliations:** 1 Psychiatry and Behavioral Sciences, Stanford University School of Medicine, USA; 2 Department of Pediatrics, Stanford University School of Medicine, USA

**Keywords:** Academic achievement, executive function, Noonan Syndrome, rasopathies, social determinants of health, socioeconomic status

## Abstract

**Objective:**

The goal of the present study is to understand whether youth with Noonan Syndrome Spectrum Disorder (NSSD) are at increased risk of neurocognitive difficulties when living in resource depleted communities.

**Method:**

Youth (5–17 years; M_age_ = 9.48 years) with NSSD (*n* = 140) and unaffected youth (4–15 years; M_age_ = 9.63 years; *n* = 85) were included. We ascertained the Child Opportunity Index Health and Environment Index (COI H/E) national-level *Z*-scores and assessed academic achievement and executive function. Multiple regressions were run to analyze the effects of diagnosis (whether the child had NSSD), COI H/E *Z*-scores, and diagnosis × COI H/E *Z*-score interaction on academic achievement (i.e., word reading, math, spelling, and sentence comprehension) and executive skills (i.e., performance-based working memory and processing speed and parent-rated measure of daily executive skills).

**Results:**

Diagnosis was a significant predictor in each model. COI H/E *Z*-score was a significant predictor of spelling and a marginally significant predictor of sentence comprehension scores. There was a significant diagnosis × COI H/E *Z*-score interaction for working memory, and marginally significant interactions for spelling and sentence comprehension scores. Higher H/E *Z*-scores were associated with better working memory in the NSSD group and better academic achievement in the unaffected group.

**Conclusions:**

While the effects of NSSD are large on all assessed domains, there is an additional burden of resource depletion on working memory abilities of youth with NSSD. Academic achievement in the NSSD group was lower than the unaffected group across resource-depleted/enriched environments, demonstrating the profound effects of NSSD on academic functioning.

## Statement of Research Significance

**Research Question(s) or Topic(s):** Are youth with Noonan Syndrome Spectrum Disorder at increased risk of the deleterious effects of community environmental resource depletion as it pertains to their neurocognitive abilities? **Main Findings:** The findings of this study are mixed. While working memory abilities seem to be lower for youth with Noonan Syndrome Spectrum Disorder when they live in resource deprived communities, academic abilities remained lower than in the unaffected group regardless of community resources. **Study Contributions:** This is the first study to examine the effects of community resources on neurocognition in Noonan Syndrome Spectrum Disorder. Our findings suggest that youth with Noonan Syndrome Spectrum Disorder from resource deprived communities should be targeted for executive skills monitoring and intervention (when indicated), but all youth with Noonan Syndrome Spectrum Disorder, regardless of community resources, should be monitored in terms of their academic achievement skills.

## Introduction

Socioeconomic status (SES) encompasses a multitude of factors, including income and parental education (which are most examined), as well as access to environmentally enriching resources. Indeed, the effects of neighborhood-level opportunity on development and neurocognitive abilities have been increasingly studied in both typically developing and clinical samples (Calub et al., 2024; Ni et al., 2022; Zhou et al., 2025). Yet, the effects of SES have been largely unexplored in youth with genetic syndromes, which is an important area of research given their increased risk of neurocognitive deficits that resource-depleted environmental conditions may exacerbate. In this paper, we examine the effects of neighborhood-level environmental quality on neurocognitive abilities in a sample of youth with Noonan Syndrome Spectrum Disorder (NSSD), which have known neurocognitive impacts, to investigate whether resource-depletion has additive predictive value on known areas of neurocognitive weakness in this population. Such research is critical, as it could point to specific community-level modifiable factors that could ameliorate outcomes in youth who are already at-risk for difficulties with neurocognitive functioning. Novel census-based measures, such as the Childhood Opportunity Index (diversitydatakids.org, 2025), allow researchers to examine such community-level factors that are likely distal influences on one’s development.

**Figure 1.**
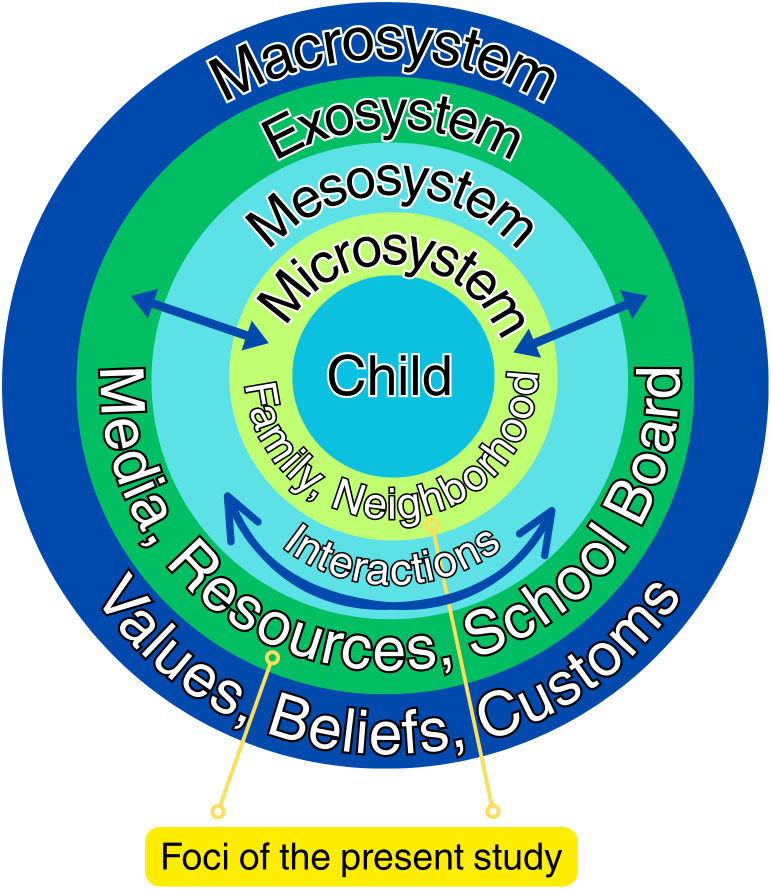
Ecological Systems Theory: Theoretical grounding for the present study. *Note.* The figure is a representation of Bronfenbrenner’s (2005) Ecological Systems Theory, with the foci of the present study demarcated. In addition to the systems illustrated, note that Bronfenbrenner also conceptualized all of the systems working with the “chronosystem,” which describes the historical time in which in an individual is living.

To better conceptualize the vast proximal and distal influences on an individual, we can use Bronfenbrenner’s Ecological Systems Theory (Bronfenbrenner, 2005) as a framework (Figure 1). At the center is the individual, in this case, a child with NSSD who is at increased risk of neurocognitive difficulties due to the individual-level factor of their genetic diagnosis. SES can be examined at the varying levels surrounding the child. For instance, in the microsystem, one could examine parental education, which has largely been the focus of the SES literature in NSSD to date (Pierpont et al., 2009, 2010). Another component of the microsystem, the focus of this study, is the neighborhood context, which remains unexplored. The mesosystem encompasses the interactions both within the microsystem and between the micro- and exosystems. The exosystem, which captures indirect influences (e.g., parental workplace, local government policies), has not yet been investigated in NSSD and is captured in the present study using the Child Opportunity Index. The macrosystem contains broader societal ideals and beliefs, for instance, the child’s country of origin’s beliefs about poverty and welfare. In sum, while there exist many proximal and distal SES influences on a child’s development, the literature, especially in NSSD, largely focuses on family factors (i.e., income, parental education; Pierpont et al., 2009, 2010). In the present study, we will expand upon the current literature by using the Childhood Opportunity Index Health and Environment Index, which allows us to better understand the influence of distal factors representing the quality of the physical neighborhood environment pollution, access to health-promoting activities and environments (e.g., walkability, extreme heat exposure, access to nature, fast food density), safety (e.g., community safety) non-profits, and health resources (e.g., insurance coverage), separate from the influence of access to educational, social, and economic resources.

Noonan Syndrome is a genetic condition with a prevalence of about 1 in 2000 births and is the most common disorder that is associated with the Ras/mitogen-activated protein kinase (RAS-MAPK) pathway (Tartaglia et al., 2011). Noonan Syndrome is associated with several genes, - the most common is *PTPN11,* followed by *SOS1* (Roberts et al., 2007; Tartaglia et al., 2002). Related conditions include individuals with *SHOC-2* variants, which cause Noonan-like syndrome with loose anagen hair (Hannig et al., 2014) and Noonan Syndrome with multiple lentigines (NSML; Gelb & Tartaglia, 2007). Individuals with *SHOC-2* variants and NSML present with somewhat similar clinical and cognitive features as individuals with Noonan Syndrome and belong to the Noonan Syndrome and Spectrum Disorders (NSSD). As such, henceforth we refer to individuals with Noonan Syndrome as well as NSML and *SHOC-2* variants as NSSD. The collection of disorders that are associated with dysregulated RAS-MAPK pathway functioning is referred to as RASopathies and includes other conditions such as neurofibromatosis type 1 (NF1) and Cardiofaciocutaneous Syndrome. This pathway is involved in many key cellular functions, such as growth, specialization, survival, and death (Aoki et al., 2008). As a result, the RAS-MAPK pathway is critical to supporting numerous neurocognitive functions, including learning and memory (Pierpont et al., 2015). Widespread neurodevelopmental areas of weakness have been identified for youth with NSSD, including delayed milestone attainment (Sharland et al., 1992), elevated symptoms of attention-deficit/hyperactivity disorder (ADHD), executive dysfunction (Naylor et al., 2023; Pierpont et al., 2015), social skills challenges (Pierpont et al., 2018), and difficulties with learning, particularly with regard to reading (Pierpont et al., 2010; Pierpont, 2016). While these neurodevelopmental concerns are quite common in youth with NSSD, intellectual capacities tend to fall within the broadly average range, and there is considerable heterogeneity across persons with NSSD with regard to the strength of verbal versus nonverbal reasoning abilities (Pierpont, 2016).

### Examinations of SES in NSSD

Prior studies of SES impacts on functioning in NSSD have been limited to examining only parental education. Pierpont and colleagues (2009) assessed family SES using parent education levels in a sample of 4–18-year-old children with NSSD. Together with hearing screening scores and manual motor dexterity, parent education significantly predicted 34% of the variability in verbal intellectual abilities in their sample (Pierpont et al., 2009). Further, parental education and motor dexterity together accounted for 40% of the variability in nonverbal intellectual abilities. In this sample, parental education did not significantly differ between children with NSSD who had familial versus sporadic mutations (Pierpont et al., 2009). Thus, the level of educational attainment of parents significantly impacts neurocognitive functioning, regardless of whether the mutation is familial or sporadic (Pierpont et al., 2009). In a combined sample of youth with NSSD and Cardiofaciocutaneous Syndrome, parental education, clinical diagnosis, age, and gestational age at birth collectively significantly predicted adaptive living skills (Pierpont et al., 2010); however, the individual contribution of parental education was not reported. Altogether, the findings of these prior studies point to SES (as measured by parental education) having an impact on intellectual abilities and adaptive living skills; however, the impact on other domains of neurocognitive abilities relevant to NSSD (i.e., academic achievement, executive function) has not been described. Thus, in the present study, we address the limitations of the prior literature by specifically examining the unique contribution of environmental factors on different domains of neurocognition above and beyond the effects of having NSSD. Further, we expand upon the conceptualization of SES by focusing on the health and environmental context, which captures the access to health-promoting environments and resources. While parental education serves as a reasonable proxy for socioeconomic influence, it is not necessarily a feasible or modifiable intervention target, nor does it capture the effects of toxic environmental exposures that could impact neurodevelopment. For instance, while a family may have socioeconomic influence related to having highly educated parents, the children in the family unit may live in an environmentally depleted area (e.g., an urban area with high levels of pollution and low access to green space) that could hinder optimal neurodevelopmental growth.

### Examinations of SES in other conditions

Research examining the effects of SES in other related populations is also sparse, but studies have identified relations with neurocognitive outcomes. For instance, SES, as measured by maternal education and parental occupation, significantly predicts IQ in children with congenital heart disease (Neukomm et al., 2022), which is relevant to NSSD given that about 80% of individuals with NSSD have a cardiovascular condition (Prendiville et al., 2014). In a European study sampling youth with NF1, lower SES (using a census-based tool similar to the Childhood Opportunity index (COI), the Index of Multiple Deprivation (Morse, 2023)) was found to be predictive of lower verbal, performance, and full-scale IQ and worse attention, hyperactivity, and social communication abilities (Geoffray et al., 2021). Another European study reported that youth with NF1 from low parental education households had significantly lower IQ scores than those from households whose parents were highly educated (Leppich et al., 2022).

### The present study

The goal of the present study is to delineate whether neurocognitive abilities (i.e., academic and executive) of youth with NSSD are more vulnerable to the effects of neighborhood-level disadvantage as compared to unaffected youth (using the Childhood Opportunity Index (COI)) by examining the interaction between diagnosis and COI Health and Environment (H/E) scores. The COI is based on 44 neighborhood indicators that were ascertained via a mixture of publicly available and proprietary information. The COI allows us to use census-tract data to better understand the level of environmental enrichment in specific neighborhoods in comparison to the local metro area, state, or nationwide data. The COI has been increasingly used in studies and has shown relations with adverse health outcomes (Tyris et al., 2025), neurocognitive and brain development in the Adolescent Brain and Cognitive Development Research Study (Zhou et al., 2025), and relations between the COI and brain activity via EEG were identified in infancy (Elansary et al., 2024).

Prior research has demonstrated negative effects of poor environmental quality, such as traffic air pollution, on academic performance and executive functioning in the general population (Gartland et al., 2022). Brain white matter, which helps support academic and executive function development (Bagautdinova et al., 2023; Clark et al., 2013), is known to be altered in NSSD (Plank et al., 2024). Thus, given that youth with NSSD are at increased risk of white matter alterations, and that white matter is related to supporting academic and executive functioning, it is hypothesized that in addition to the effects of diagnosis and COI H/E scores, there will be significant diagnosis × COI H/E interaction effects in the executive and academic achievement models. Understanding whether youth with NSSD have increased vulnerability to the effects of neighborhood-level disadvantage is imperative, as the findings (1) may implicate further neurocognitive functioning and SES relations that have been documented in the general population (Zhou et al., 2025), but are largely unexplored in genetic syndromes and (2) could demonstrate that there is a need for more targeted, rigorous, and earlier intervention for youth with NSSD as a function of their neighborhood environmental quality. The present study will build upon prior SES research in NSSD (Pierpont et al., 2009, 2010) by examining a specific facet of advantage: the quality of the local environment in terms of accessibility and toxins (i.e., the COI Health and Environment Index). Additionally, in contrast to earlier work focusing on intellectual and adaptive living skills, the present study examines an important domain of ability, academic achievement, that has yet to be explored in this context in NSSD.

## Method

### Participants

Participants (4–17 years) were seen in the context of two broader behavioral and neuroimaging phenotyping studies of youth with NSSD (McGhee et al., 2024; Naylor et al., 2023). Participants included youth with NSSD (*n =* 140, female *n* = 74, Mean_age_ = 9.48, SD_age_ = 3.09) and unaffected youth (*n =* 85, female *n* = 63, Mean_age_= 9.73, SD_age_ = 2.60). Unaffected participants were recruited via advertisements in local schools, community centers, and social media posts. Participants with NSSD were primarily recruited via social media posts and patient organization groups. Participants from Wave 1 (2013–2020) completed the Wechsler Intelligence Scales for Children – 4^th^ Edition or Wechsler Preschool and Primary Scale of Intelligence – 3^rd^ Edition (Wechsler, 2002, 2003), the Wide Range Achievement Test 4^th^ Edition (WRAT; Wilkinson & Robertson, 2006, 2017), and the Behavior Rating Inventory of Executive Function Preschool or 2^nd^ Edition (BRIEF-P/2; Gioia et al., 2015, 2003). One participant was excluded from Wave 1 because the participant did not complete the Working Memory or Processing Speed Indices, which are included in the present analyses. Participants from Wave 2 (2021–2025) completed the WRAT-5 and the BRIEF-2. Exclusion criteria for all youth included (a) an IQ criterion (unaffected group IQ > 80 to reduce the likelihood that individuals with neurodevelopmental concerns would be in the comparison group; NSSD group IQ ≥ 70) (b) gestational age ≤ 34 weeks, (c) low birth weight (<2000 g), (d) presence of neurological or severe psychiatric illness, (e) sensory deficits that preclude participation in assessments, (f) history of significant head trauma with loss of consciousness, g) history of drug and/or alcohol use, (h) presence of a neurological disorder known to affect cognitive development or brain structure; (i) known presence of gliomas in the cerebellum, brainstem, or basal ganglia. All participants with NSSD provided proof of genetic testing. Unaffected youth were excluded if they had a formal neurodevelopmental disorder diagnosis. A summary of mutation types represented in the sample are in Table 1. The study was conducted in accordance with procedures outlined in the Helsinki Declaration, as was approved by the Stanford University School of Medicine Institutional Review Board. Table 2 describes the participant demographics.

**Table 1.** Mutation types represented in NSSD sample

Genetic mutation	*n* (%)
*PTPN11*	94 (67.1)
*SOS1*	21 (15.0)
*NSML*	8 (5.7)
*NRAS*	3 (2.1)
*RAF1*	3 (2.1)
*RIT1*	3 (2.1)
*KRAS*	2 (1.4)
*SOS2*	2 (1.4)
*LZTR1*	2 (1.4)
*SHOC-2*	2 (1.4)

**Table 2.** Sample demographics

	NSSD	Unaffected	*p*
Wave 1 *n* (%)	41 (29.3)	85 (100.0)	<0.001
Wave 2 *n* (%)	99 (70.7)	0 (0.0)	
Total *n*	140	85	
Ethnicity			0.006
Hispanic or Latino	9 (6.4%)	15 (17.6%)	
Not Hispanic or Latino	130 (92.9%)	62 (72.9%)	
Missing	1 (0.7%)	8 (9.4%)	
Race			0.048
Asian	4 (2.9%)	4 (4.7%)	
African American	4 (2.9%)	0 (0.0%)	
White	120 (85.7%)	56 (65.9%)	
Bi/Multiracial	10 (7.1%)	11 (12.9%)	
Declined to state	1 (0.7%)	3 (3.5%)	
Missing	1 (0.7%)	11 (12.9%)	
Maternal education level			0.425
Junior High School	1 (0.7%)	0 (0.0%)	
High School Graduate	2 (1.4%)	1 (1.2%)	
Partial College	24 (17.1%)	12 (14.1%)	
College Graduate	61 (43.6%)	28 (32.9%)	
Graduate Degree	52 (37.1%)	40 (47.1%)	
Missing	0 (0.0%)	4 (4.7%)	
Neurocognitive Measures (M(SD))			
BRIEF Version			–
Preschool Edition	5 (3.6%)	7 (8.2%)	
Second Edition	134 (95.7%)	69 (81.2%)	
Missing	1 (0.7%)	9 (10.6%)	
Performance Based Measures			–
WPSSI-III (4-7 years)	6 (4.3%)	8 (9.4%)	
WISC-IV	35 (25.0%)	77 (90.6%)	
BRIEF Global Executive Composite (*n* = 215)	63.8 (11.9)	48.1 (9.5)	<0.001
Working Memory Index (*n* = 111)	90.8 (14.1)	104.0 (13.5)	<0.001
Processing Speed Index (*n* = 124)	87.9 (15.0)	101.0 (14.3)	<0.001
WRAT Word Reading (*n* = 124)	96.1 (15.6)	118.0 (130.0)	<0.001
WRAT Math (*n* = 171)	89.0 (16.1)	112.0 (11.4)	<0.001
WRAT Spelling (*n* = 164)	88.2 (14.6)	112.0 (13.2)	<0.001
WRAT Sentence Comprehension (*n* = 138)	97.2 (16.3)	121.0 (15.9)	<0.001
Childhood Opportunity Index (COI)			
Health and Environment Index			
*Z*-Score Mean (SD)	0.496 (0.474)	0.659 (0.442)	0.012
*Z*-Score Range	[−1.24, 1.55]	[−0.853, 1.32]	
Percentile Mean (SD)	62.7 (25.7)	71.2 (24.8)	0.012
Percentile Range	[3.00, 100]	[7.00, 100]	
Overall COI			
* Z*-Score Mean (SD)	0.632 (0.663)	1.07 (0.826)	<0.001
* Z*-Score Range	[−0.733, 2.18]	[−0.344, 2.47]	
Percentile Mean (SD)	68.9 (23.7)	79.1 (22.4)	<0.001
Percentile Range	[13.0, 100]	[28.0, 100]	

*Note.* NSSD = Noonan Syndrome Spectrum Disorder; Fisher’s Exact Tests were used to test for significant differences on categorical variables, while Mann–Whitney *U*-Tests were used to test for significant differences on numerical variables.

### Measures

*Child Opportunity Index.* The Child Opportunity Index (diversitydatakids.org, 2025) is a publicly available database that uses neighborhood-level data to rank addresses on various aspects of opportunity. The present study used the Child Opportunity Index 3.0 data. We focus our analyses on the Health and Environment Index, which encompasses the quality of the environment (e.g., pollution, airborne microparticles, extreme heat exposure), safety (e.g., number of community safety-related non-profits), and health resources (insurance coverage) available in a specific location. *Z*-scores (*M* = 0; *SD* = 1) were used, with higher scores indicating greater advantage. Supplementary analyses include the overall COI Index *Z*-scores, which in addition to the Health and Environmental Index, also includes the Education and Social and Economic Indices.

*Wide Range Achievement Test-4* ^*th*^ *or 5* ^*th*^ *Edition (WRAT).* The WRAT (Wilkinson et al., 2006, 2017) is a performance-based measure of academic achievement for individuals 5 years or older, including tasks assessing word reading, mathematical computations, spelling, and sentence reading comprehension. The ascertained scores are age-referenced standard scores with *M* = 100, *SD* = 15 (higher scores reflecting stronger performance).

*Behavior Rating Inventory of Executive Function-2* ^*nd*^ *(BRIEF-2) or Preschool Edition (BRIEF-P).* The BRIEF-2 /P (Gioia et al., 2015, 2003) are caregiver-rated measures of daily executive functioning skills. The BRIEF-P (*n* = 12) is normed for children 2–5:11 years and the BRIEF-2 (*n* = 203) is normed for youth 5–18 years old. In the present analyses, we used the Global Executive Composite, which encompasses all scales on the overall measure, and thus reflects a conglomerate of a broad range of daily executive functioning behaviors (e.g., self-monitoring, organizational skills, inhibition). The indices are age-referenced *T*-scores with *M* = 50, *SD* = 10, with higher scores indicating more executive dysfunction.

*Wechsler Intelligence Scales for Children-4* ^*th*^ *(WISC-IV) Edition or Wechsler Preschool and Primary Scale of Intelligence-3* ^*rd*^ *(WPPSI-III) Edition*. Wave 1 participants (NSSD group *n* = 35, unaffected group *n* = 75). Completed the Wechsler scales (Wechsler, 2002, 2003) as part of their research participation (WPPSI-III *n* = 12; WISC-IV *n* = 112). The Working Memory (WMI) and Processing Speed (PSI) Indices were ascertained from the Wechsler scales (Wechsler, 2002, 2003) for these participants. The indices are age-referenced standard scores with *M* = 100, *SD* = 15, with higher scores indicating better performance.

### Analytic strategy

Multiple regression models were run to predict academic achievement variables (i.e., WRAT Math, Reading, Spelling, and Sentence Comprehension) and executive functioning variables (i.e., Wechsler WMI and PSI scores and BRIEF Global Executive Composite). Predictors included diagnosis (i.e., whether the child had Noonan Syndrome or was unaffected), COI H/E Z-scores, and a diagnosis × COI H/E Z-score interaction term. Partial eta squared was calculated for the effect size of each predictor (0.01 = small effect, 0.06 = medium effect, 0.14 = large effect). Regression models were corrected using a Bonferroni correction; corrected *p*-values are presented.

## Results

### Academic achievement

**Figure 2.**
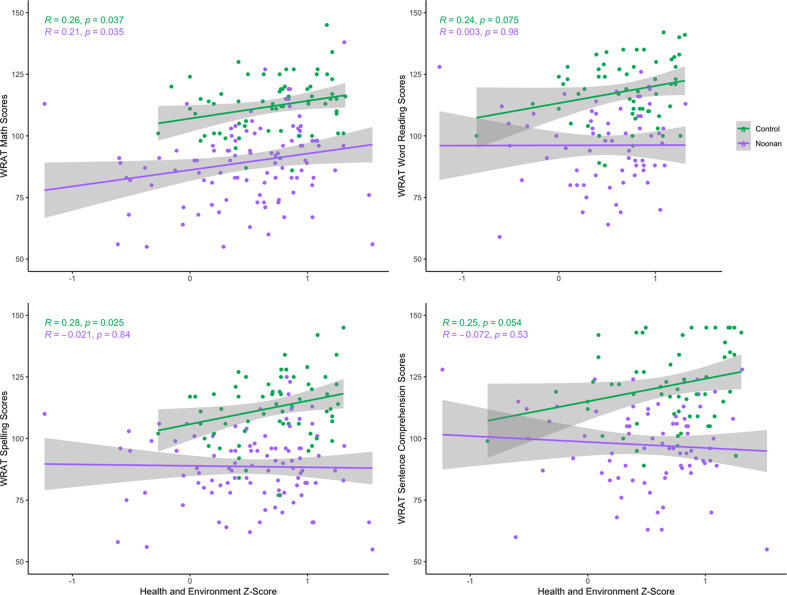
Correlations between academic achievement measures and health and environment *Z*-scores.

For WRAT academic measures, all models were statistically significant (Math: *F*(3, 167) = 38.59, *p* < 0.001, R^2^ = 0.398; Word Reading: *F*(3, 120) = 24.89, *p* < 0.001, R^2^ = 0.368; Spelling: *F*(3, 160) = 40.42, *p* < 0.001, *R* ^2^ = 0.420; Sentence Comprehension: *F*(3, 134) = 26.99, *p* < 0.001, R^2^ = 0.362) (Figure 2). Diagnosis was a significant predictor in each model with large effects (*p* < 0.001, partial η^2^ = 0.36–0.42); in each model, youth with NSSD had poorer academic functioning than unaffected youth. H/E *Z*-scores were significant predictors of Spelling scores (*p* = 0.037, partial η^2^ = 0.008): every 1 H/E *Z*-score increase was associated with a 9.47 standard score increase in Spelling scores (*B* = 9.47). H/E *Z*-scores were also marginally significant predictors of Sentence Comprehension scores (*p* = 0.058, partial η^2^ = 0.003): every 1 H/E Z-score increase was associated a 9.20 standard score increase in Sentence Comprehension scores. H/E *Z*-scores were not significant predictors of Math (*p* = 0.096) or Word Reading scores (*p* = 0.110). The diagnosis × H/E *Z*-score interaction was marginally significant in predicting Spelling (*p* = 0.058, partial η^2^ = 0.02) and Sentence Comprehension scores (*p* = 0.059, partial η^2^ = 0.03). For the marginally significant interaction effects, higher H/E *Z*-scores were associated with stronger Spelling and Sentence Comprehension scores in the unaffected group, while the NSSD group’s slope was nearly flat (i.e., negligible association between academic achievement and H/E *Z*-scores). A follow-up analysis, detailed in the Supplement, was conducted to examine whether the effects of the overall COI on academic achievement were the same as the H/E Index. Similar to the H/E Index findings, there was a significant effect of diagnosis in all models. Contrastingly, however, there were significant effects of the overall COI *Z*-scores on all academic achievement measures. There were no significant interaction effects.

### Executive function

**Figure 3.**
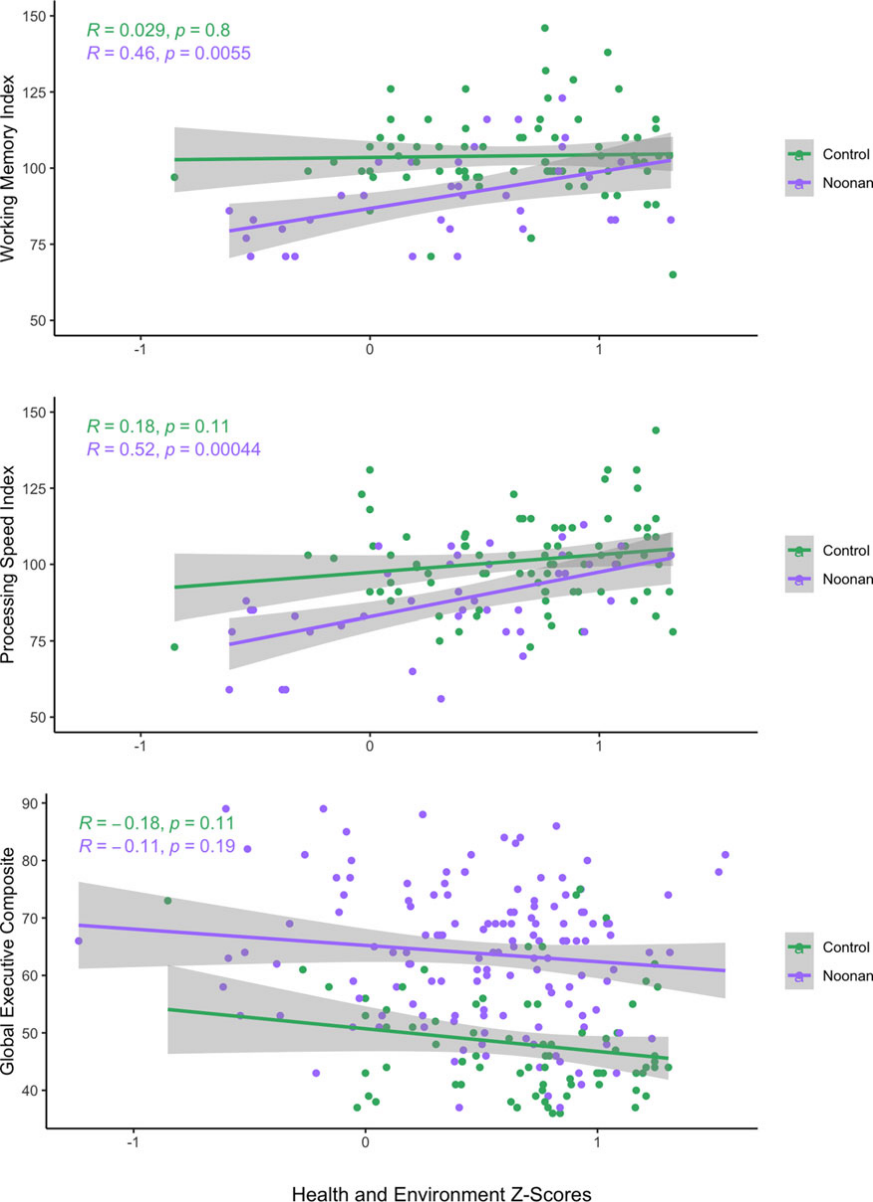
Correlations between executive function measures and health and environment *Z*-scores.

The regressions models for Wechsler WMI (*F*(3, 107) = 10.61, *p* < 0.001, R^2^ = 0.207)), PSI (*F*(3, 120) = 13.85, *p* < 0.001, R^2^ = 0.238), and Global Executive Composite scores (*F*(3, 211) = 34.55, *p* < 0.001, R^2^ = 0.319) were significant (Figure 3). For each regression, diagnosis was a significant predictor with a large effect size (*p* < 0.001, partial η^2^ = 0.18–0.32); having NSSD was associated with lower executive function scores. For the WMI, there was a significant diagnosis × H/E *Z*-score interaction effect (*p* = 0.042, partial η^2^ = 0.04): higher H/E *Z*-scores were associated with stronger working memory in the NSSD group, while for the unaffected group, the slope was nearly flat.

A follow-up analysis, expanded upon in the Supplement, was conducted to examine whether the effects of the overall COI on executive function were the same as the H/E Index. Diagnosis was a significant predictor in all models. The overall COI was a significant predictor of PSI, but not WMI or GEC scores. There were no significant interaction effects.

### Follow-up exploratory analyses

Follow-up exploratory Pearson correlations were conducted to examine which subscales of the H/E index were related to neurocognitive variables that were significantly or marginally predicted by the H/E index alone or in an interaction (i.e., Wechsler WMI, WRAT Spelling, Sentence Comprehension) in the NSSD and unaffected samples as separate groups. Supplemental Figure 3 depicts these correlations. In the NSSD group, Safety-Related Resources were significantly related to the Wechsler WMI *(r = 0.388*, *p* = 0.021). In the unaffected group, Health Resources were significantly related to Spelling (*r* = 0.337, *p* = 0.006).

A sensitivity analysis was conducted to examine the effects of maternal education (i.e., a proximal, family-centered variable) on academic achievement and executive function. In brief, there were no significant effects of maternal education on executive functioning variables, but there were significant effects of maternal education on all academic achievement variables. There were no diagnosis × maternal education interactions on any variables. This analysis is further detailed in the Supplement.

## Discussion

To our knowledge, this is the first study to examine the effects of the environment and health access on cognition and academic functioning in NSSD. Furthermore, the present study expands upon the prior literature by examining SES influences on novel domains in NSSD: executive and academic abilities. Our findings revealed mixed results. As expected, diagnosis was a significant predictor in all models, while the H/E *Z*-score was a significant predictor only for spelling, and was a marginally significant predictor of sentence comprehension abilities. There was a significant interaction between diagnosis and COI H/E scores on performance-based working memory scores. In this model, the slope of the unaffected group was relatively flat, while the slope of the NSSD group was positive. This suggests that, in line with our hypothesis, youth with NSSD living in resource-depleted areas are at increased risk of difficulties with working memory as compared to unaffected youth but also as compared to youth with NSSD from resource enriched areas. In other words, as it pertains to working memory abilities, there is an additive adverse impact of environmental resource depletion for youth with NSSD. Importantly, this interaction was not significant in our analysis of the overall COI, signifying a unique relationship between health and environmental factors with working memory abilities, that is obscured when examining the COI as a whole (i.e., with education and social/economic factors included). While not statistically significant, there appears to be a similar pattern in the data for the relationship between health and environmental factors with processing speed scores (Figure 2). It is possible that underlying developmental white matter alterations, which are present in NSSD (Plank et al., 2024), are being further exacerbated by deleterious environmental effects captured by the H/E index, thereby negatively impacting downstream executive abilities. Although research has demonstrated a relation between the COI and brain development (Elansary et al., 2024; Zhou et al., 2025), further neuroimaging research is needed to better understand these impacts in NSSD.

Interestingly, the inverse was true for academic measures. In models with marginally significant interaction effects (i.e., spelling and sentence comprehension), the slope of youth with NSSD was largely flat, while the slope of the unaffected group was positive. Thus, while the unaffected group’s performance increased in more enriched environments, consistent with large population studies (Zhou et al., 2025), youth with NSSD’s performance was largely similar. It could be the case that the effect of the NSSD phenotype on difficulties with academic abilities is so strong and central to the phenotype that it would be difficult to find a statistically significant effect of more environmental factors on these abilities. Additional analyses examining the overall COI Index generally mirrored those of the H/E Index; while there was a significant effect of the overall COI in each model, there were no significant interaction effects. Further, the slopes of the academic domains × overall COI for NSSD groups were largely flat (Supplemental Figure 1). These additional results underscore the significant impacts of NSSD on academic functioning, regardless of broader socioeconomic advantage.

The present findings yield important information regarding the influences of neurocognitive difficulties in youth with NSSD. In particular, our contradictory findings point to different avenues and priorities for intervention in youth with NSSD. For performance-based executive skills, it may be imperative to target youth from resource-depleted environments for screening and subsequent interventions. For academic skills, the effect of NSSD on learning abilities is seemingly so strong, that environmental enrichment is largely noncontributory. Thus, it is important that all youth with NSSD be screened for difficulties with academic abilities, and when relevant, referred for interventions. Although the efficacy and effectiveness of academic interventions have not been specifically tested in NSSD, in addition to traditional instructional methods (e.g., Orton-Gillingham method for dyslexia, strategy instruction for dyscalculia), there has been a push to create more personalized, reinforcement learning based activities (potentially with artificial intelligence) to facilitate academic learning in youth with genetic disorders (Stasolla et al., 2024).

While interventions can be delivered in the microsystem, such as school-based interventions that have demonstrated gains in attention an executive function (Paananen et al., 2022), our findings underscore the opportunity for change at the exosystem level. Specifically, our findings point to the importance of advocacy, systemic-level change, and community-level interventions to ameliorate outcomes in youth. In particular, our exploratory correlations yielded significant correlations with Safety (e.g., housing, availability of safety-related non-profits) and Health Related Resources (e.g., insurance coverage, availability of health-related non-profits). Increasing access to these resources may have positive effects on the development of youth, particularly youth with NSSD. Prior research has demonstrated such endeavors are feasible and effective. For instance, community-based interventions targeting play and reading skills in early childhood have been demonstrated to be effective in middle- and low-income countries (Maulik & Darmstadt, 2009). Further, a review conducted by Komro and colleagues (2013) demonstrated how evidence-based strategies at the community-level (e.g., child health care access, living wage ordinances, urban design policies) have significant effects on optimal child neurocognitive, social/emotional, and physical health development. These are tangible, reproducible interventions that can be advocated for by practitioners, researchers, educators, and families to ameliorate outcomes in youth. Of relevance to the present findings, this could include: (1) local policy changes to improve adequate housing access for all youth; (2) expanded health insurance coverage (which is critical for youth with chronic health conditions, as is the case for youth with NSSD); (3) urging local reform to improve child safety; and (4) encouraging the development of free/low-cost health-related services. In order to accomplish these, it is critical that researchers and practitioners, as influential community members, advocate for these public health endeavors, and in the meantime, connect their patients with these resources when they are available.

The findings of the present study build on prior work conducted in NSSD demonstrating a relation between socioeconomic factors and cognition in youth. Pierpont and colleagues (2009) reported a relation between parental education levels and verbal intellectual abilities, while our sample found marginally significant interaction effects between the COI H/E scores and verbally mediated academic abilities (i.e., spelling, sentence comprehension). These converging findings align with the Ecological Systems framework in that both microsystemic factors (i.e., parental education) and exosystemic factors (i.e., community resources) are related to the development of verbal skills in youth with NSSD, in addition to individual level factors (i.e., risk of neurocognitive difficulties as a result of genetic diagnosis).

### Limitations and future directions

Although the present sample of youth with NSSD is large compared to what is typically published, the sample was not large enough to examine within-group differences in the NSSD group by gene mutation type. Our sample was not fully representative. The unaffected youth in our study came from more advantaged backgrounds than average and had high academic achievement, a common issue in university-based research. Future studies should replicate these findings in other geographic areas with different population characteristics. Further, the present study is a cross-sectional study, which precludes interpretations of causality and directionality. Indeed, future research is needed to examine the cumulative effects of socioeconomic (dis)advantage in NSSD by implementing longitudinal designs. Unaffected adult samples, for example, have demonstrated that lead has cumulative health effects in adulthood (Shih et al., 2007). Finally, the present study examined the effects of the environmental quality (e.g., pollutants, safety, and healthcare coverage) on functioning. Given that SES is multifaceted and can be studied at varying levels, future research should examine other aspects of SES and its effects on outcomes in NSSD. For instance, little is known about macrosystemic level factors on the development of youth with NSSD, which would indeed necessitate international, multi-site collaborations.

## Conclusions

To our knowledge, this is the first study to examine the effects of health and environmental resources on functioning in youth with NSSD. Our findings demonstrated that resource depletion is significantly related to working memory abilities in youth with NSSD, and there was emerging evidence of relations with verbally mediated academic skills. While the effect of the NSSD phenotype was evident on all of assessed abilities, the evidence of the impact of environmental resource depletion on several domains is compelling and indicates a greater need for advocacy, support, and change in marginalized communities.

## Supporting information

10.1017/S1355617726101969.sm001 Pardej et al. supplementary material Pardej et al. supplementary material

## Supplementary material

The supplementary material for this article can be found at https://doi.org/10.1017/S1355617726101969.
