# Supplementary material for: Effects of neighborhood level health and environment quality on academic and executive abilities in youth with Noonan syndrome spectrum disorder
Source: J Int Neuropsychol Soc. 2026 May 6:1–9. Online ahead of print. doi: 10.1017/S1355617726101969 (PMC13182281; doi:10.1017/S1355617726101969)
Supplement: Pardej et al. supplementary material [file S1355617726101969sup001.pdf]

Supplement

Regression Models Examining the Effects of the Overall COI on Academic and Executive Abilities

The effects of the overall COI on academic and executive measures were investigated using the same statistical analysis plan as described in the main text for the Health and Environment Index. For WRAT academic measures, all models were statistically significant [Math: $F(3, 167)=39.91$, $p<.001$, $R^2=.407$; Word Reading: $F(3, 120)=26.28$, $p<.001$, $R^2=.381$; Spelling: $F(3, 160)=41.97$, $p<.001$, $R^2=.429$; Sentence Comprehension: $F(3, 134)=29.82$, $p<.001$, $R^2=.387$]. Diagnosis was a significant predictor in all models, with large effects (partial η^2's=.37-.43). There was a significant, medium effect of the overall COI on Math scores ($p=.018$, partial $\eta^2=.07$). Every 1 COI Z-score increase was associated with a 5.03 standard score increase in Math scores (B=5.03). There was a significant, small effect of the overall COI on Word Reading scores ($p=.032$; partial $\eta^2=.03$). Every 1 COI Z-score increase was associated with a 5.06 standard score increase in Word Reading scores (B=5.06). There was a significant, small effect of the overall COI on Spelling scores ($p=.017$, partial $\eta^2=.04$). Every 1 overall COI Z-score increase was associated with a 5.23 standard score increase in Spelling scores (B=5.23). There was a significant, small effect of the overall COI on Sentence Comprehension scores ($p=.003$, partial $\eta^2=.05$). Every 1 COI Z-score increase was associated with a 7.78 standard score increase in Sentence Comprehension scores (B=7.78). There were no significant diagnosis x COI interaction effects for any academic achievement outcomes [Math $p=.937$; Word Reading $p=.324$; Spelling $p=.298$; Sentence Comprehension $p=.082$].

All executive models were statistically significant [Wechsler Working Memory Index (WMI) $F(3, 107)=8.89$, $p<.001$, $R^2=.177$; Wechsler Processing Speed Index (PSI) $F(3,$

120)=10.24, $p<.001$, $R^2=.184$; BRIEF Global Executive Composite $F(3, 211)=32.84$, $p<.001$, $R^2=.308$]. Diagnosis was a significant predictor in all models, with large effects (partial $\eta^2=.17$-.32). There was a significant, small effect of the overall COI on PSI scores ($p=.046$, partial $\eta^2=.05$). Every 1 COI Z-score increase was associated with a 3.81 standard score increase in PSI scores (B=3.81). There were no significant effects of the overall COI on WMI ($p=.297$) or GEC scores ($p=.816$). There were no significant diagnosis x COI interaction effects in any of the executive functioning models ($p=.055$-.820).

Supplemental Figure 1. Association Between Academic Achievement Measures and Childhood Opportunity Index Z-Scores

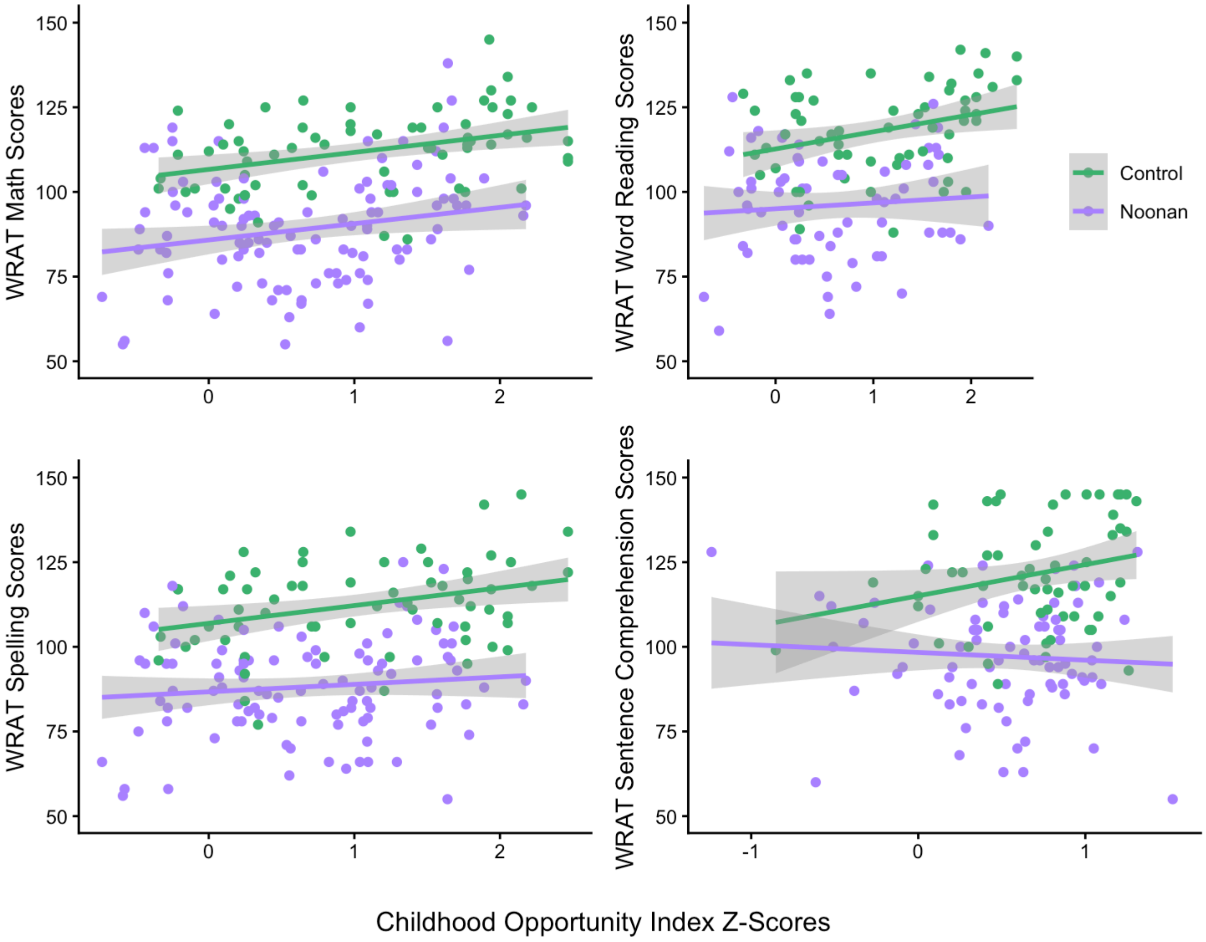

Supplemental Figure 2. Association Between Executive Function Measures and Childhood Opportunity Index Z-Scores

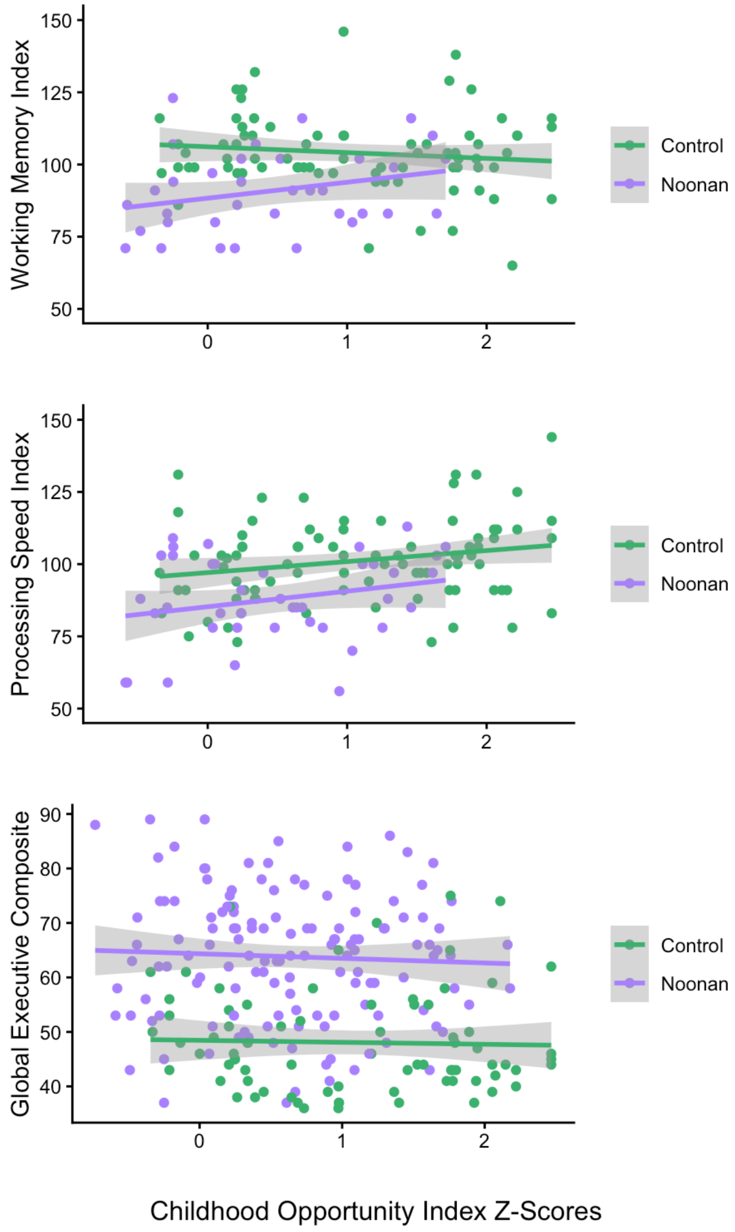

Supplemental Figure 3. Correlations between Childhood Opportunity Index Health and Environment Index Subscales with Performance Across Youth with Noonan Syndrome and Unaffected Controls

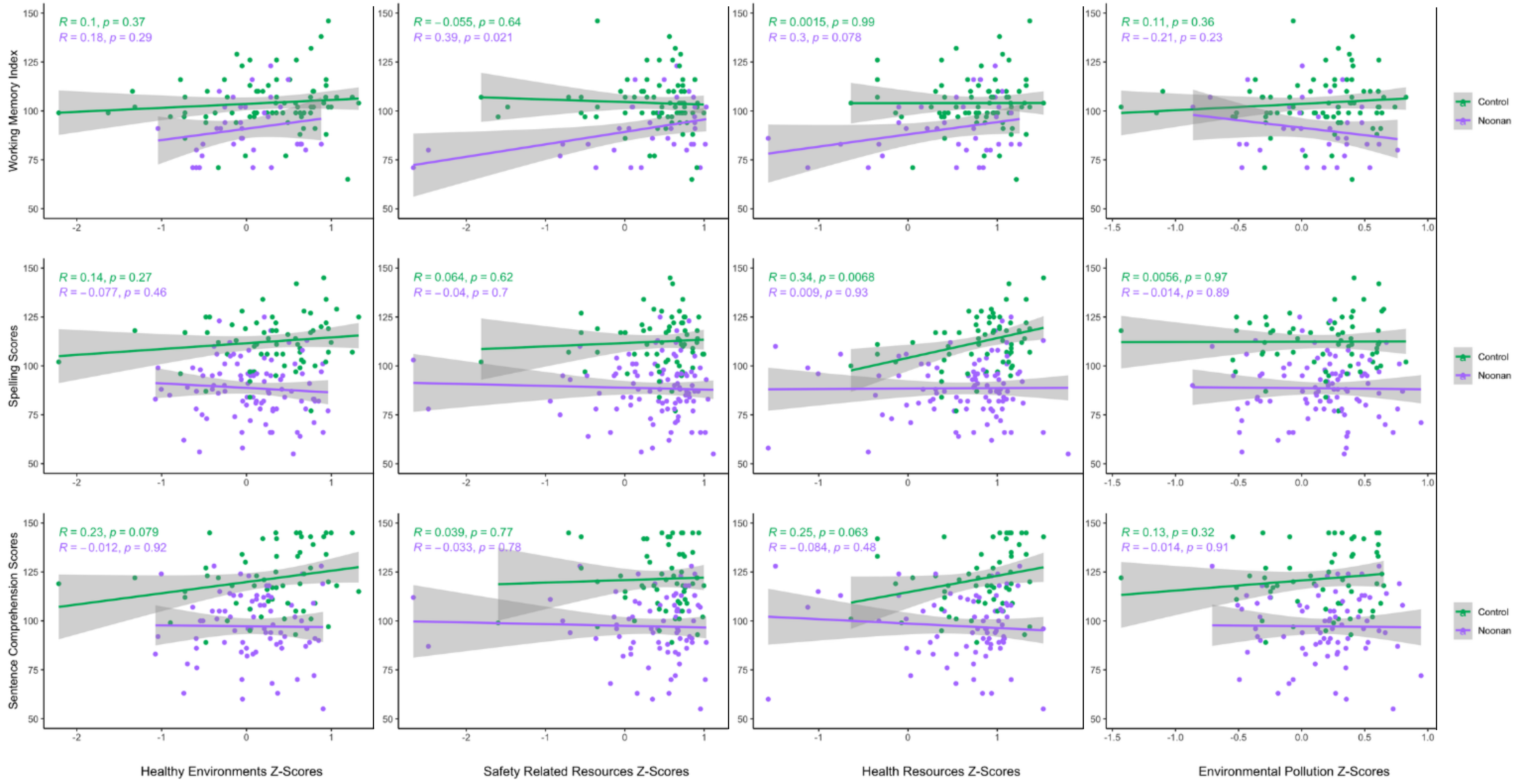

Figure Description: Exploratory Pearson correlations were run to examine which subscales of the Childhood Opportunity Index Health and Environment Index were likely most contributory to significant or marginally significant models in which the Health and Environment Index was a significant or marginally significant predictor of performance.

Sensitivity Analysis Examining Maternal Education

A sensitivity analysis was conducted using maternal education as a proximal, family-centered variable. Given that maternal education is a categorical variable, ANOVA, rather than multiple regression, was used. The overall results of the ANOVAs are in Supplemental Tables 1 and 2. There was no significant effect of maternal education on executive function variables (Wechsler WMI p=.402, Wechsler PSI p=.072, BRIEF GEC p=.197**)** or a diagnosis x maternal education interaction (Wechsler WMI p=.776, Wechsler PSI p=.616, BRIEF GEC p=.059). There was a significant effect of maternal education on WRAT Word Reading, Sentence Comprehension, Math, and Spelling scores (p=.002, .006, .013, .016 respectively), but no diagnosis x maternal education interaction effects (p=.695, .834, .823, .282, respectively).

Supplemental Table 1. Results of Sensitivity Analysis Examining Executive Function Variables

	F	*p*
Model Predicting Wechsler Working Memory Index		
Diagnosis	24.70	<.001
Maternal Education	0.70	.402
Diagnosis x Maternal Education	0.08	.776
Model Predicting Wechsler Processing Speed Index		
Diagnosis	26.03	<.001
Maternal Education	2.39	.072
Diagnosis x Maternal Education	0.59	.616
Model Predicting Global Executive Composite		
Diagnosis	99.83	<.001
Maternal Education	1.57	.197
Diagnosis x Maternal Education	2.51	.059

Supplemental Table 2. Results of Sensitivity Analysis Academic Achievement Variables

	F	*p*
Model Predicting WRAT Word Reading		
Diagnosis	76.16	<.001
Maternal Education	5.15	.002
Diagnosis x Maternal Education	.364	.695
Model Predicting WRAT Sentence Comprehension		
Diagnosis	79.00	<.001
Maternal Education	4.29	.006
Diagnosis x Maternal Education	0.18	.834
Model Predicting WRAT Spelling		
Diagnosis	115.04	<.001
Maternal Education	3.50	.016
Diagnosis x Maternal Education	1.27	.282
Model Predicting WRAT Math		
Diagnosis	102.70	<.001
Maternal Education	3.68	.013
Diagnosis x Maternal Education	0.30	.823
